# Gastric Dilatation and Tetany

**Published:** 1904-05-21

**Authors:** 


### GASTRIC DILATATION AND TETANY.

A case of severe gastric tetany successfully treated by gastro-jejunostomy is recorded by Dr. J. H. Cunningham,[1] who proceeds to summarise the chief features of the affection and the results of various methods of treatment. His case was that of a man 28 years old, who first received hospital treatment in January 1902. He had then been suffering from recurring attacks of vomiting for six years. During these attacks, which lasted one or two weeks, there was constant epigastric distress and feeling of illness, and very little food could be taken on account of the sickness, which larger quantities induced. For a short time previously to his admission to hospital he had noticed tingling of the hands which came on after each severe attack of vomiting, and continued for about half an hour. He remained in hospital for a fortnight and was much relieved by the medical treatment to which he was subjected. It was noted, however, that every attempt to pass a stomach tube failed, and was followed by tingling in the hands and contractions of the fingers. The improvement did not last long, the vomiting recurred, while the tingling and numbness in the hands became more pronounced, and there was rapid loss of weight. In March 1902 he again became an in-patient and during his stay had several attacks of tetany, one especially severe fit following an unsuccessful endeavour to pass a stomach tube. Examination of the vomit showed the presence of free hydrochloric acid and the absence of lactic acid. The urine showed a slight trace of albumen and many hyaline and granular casts were present. The stomach was considerably dilated and peristaltic movements were visible. Surgical treatment was advised but was refused by the patient, who was discharged at the end of three weeks much improved in general health.

Nine months later a severe attack of tetany supervened, the patient being unconscious for four consecutive days. An operation was again recommended but was refused. Six months later, in October of last year, the patient was once more admitted to hospital on account of a severe tetanic seizure. On this occasion he agreed to undergo an operation. He was anæsthetised accordingly, a stomach tube was passed and the stomach washed out with normal salt solution. This was the first occasion on which the attempt to pass a tube was successful. A posterior gastro-jejunostomy was performed, pyloric obstruction being found to be present. Convalescence was uneventful, and 12 days later the patient was taking stewed meat and potatoes. From this time on he remained free from all gastric symptoms, and by March of this year had gained

42 lbs. in weight. When the patient was last seen the stomach was of proper size, and the urine was normal in all respects.

In commenting on this case Dr. Cunningham remarks that the condition of the urine during the attacks of vomiting and tetany must be in favour of the supposition that some toxic substance was acting upon the kidney. This would be in accordance with the theory put forward by Bouveret and Devic, who believe that tetany is due to the absorption of toxic material formed in a dilated stomach in which there is prolonged retention of food. The fact that the convulsive seizures usually follow an attack of vomiting or the passage of a stomach tube, would, under the above theory, find its analogy in strychnine poisoning in the frog and in tetanus where convulsions may be induced by the application of mechanical stimuli.

With regard to the prognosis of gastric dilatation with tetany, statistics show that recovery without operation is comparatively rare. Recorded cases, not treated surgically, show a fatal issue in nearly 80 per cent., while the recorded cases treated by operation have a mortality of about 38 per cent. In the ordinary course of events death does not ensue until some time after the seizures have become well established, although instances are known in which the patient died within a few hours of a first convulsive attack. In view of the better results obtained by operative interference, Dr. Cunningham insists that those cases of gastric dilatation which develop tetany as a sequence should be operated upon as soon as possible after the onset of such symptoms.

[1] Annals of Surgery, April.

